# The Protective Effects of Mitochondrial Therapy Against Vincristine- Induced Nephrotoxicity in the Rat’s Renal Proximal Tubular Cells

**DOI:** 10.5812/ijpr-159628

**Published:** 2025-02-10

**Authors:** Armaghan Lohrasbi, Abdollah Arjmand, Farzaneh Kamranfar, Mehdi Aghsami, Jalal Pourahmad

**Affiliations:** 1 School of Pharmacy, Iran University of Medical Sciences, Tehran, Iran; 2 Department of Pharmacology and Toxicology, School of Pharmacy, Shahid Beheshti University of Medical Sciences, Tehran, Iran; 3 Department of Pharmacology and Toxicology, School of Pharmacy, Iran University of Medical Sciences, Tehran, Iran

**Keywords:** Vincristine, Oxidative Stress, Nephrotoxicity, Mitochondria Transplantation, Renal Proximal Tubular Cells

## Abstract

**Background:**

The application of vincristine (VCR) in treating a range of cancers is well-documented, showcasing its considerable effectiveness. Nevertheless, its clinical application is constrained by its impact on healthy tissues and various organ systems. Specifically, it can compromise kidney function, resulting in toxicological concerns. Studies have demonstrated that vincristine contributes to nephrotoxicity via the induction of oxidative stress.

**Objectives:**

The present research focused on assessing the influence of mitochondrial transplantation in mitigating the mitochondrial and cellular toxicity associated with VCR in renal proximal tubular cells (RPTCs).

**Methods:**

This investigation evaluated specific toxicity metrics, including cell death, reactive oxygen species (ROS) generation, decreased mitochondrial membrane potential (MMP), glutathione (GSH) concentration, succinate dehydrogenase (SDH) activity, lipid peroxidation (LPO), and adenosine triphosphate (ATP) levels. Freshly prepared active mitochondria were obtained from the kidneys of Wistar rats.

**Results:**

The data demonstrated the cytotoxic effects of VCR on RPTCs. It was further observed that vincristine triggered oxidative stress, characterized by heightened ROS levels, diminished GSH content, decreased SDH activity, and increased lipid peroxidation. Furthermore, VCR caused notable damage to both mitochondrial and lysosomal membranes, along with a significant decrease in ATP content. The innovative strategy of mitochondrial transplantation mitigated oxidative stress, alleviated mitochondrial membrane damage, and prevented ROS-mediated apoptosis signaling induced by vincristine in RPTCs. Our results also indicated an increase in ATP levels within these cells.

**Conclusions:**

Our investigation suggests that the proposed treatment modality may prove beneficial in addressing drug-induced nephrotoxicity.

## 1. Background

Vincristine (VCR), a vinca alkaloid, is a common chemotherapy medication used to treat an array of cancers, including leukemia, lymphoma, and Hodgkin's disease. Approximately 8% to 15% of VCR is excreted in the urine as an unchanged parent drug, indicating the susceptibility of the kidneys to the cytotoxic effects of VCR. It is assumed that 25% of cardiac output is directed to the kidneys, rendering them one of the most susceptible organs to the toxicity of chemical therapeutics. Additionally, their crucial role in the excretion and elimination of numerous substances exposes them to various toxicants. Therefore, nephrotoxicity has become a major limiting factor in using VCR as an antineoplastic agent (1).

Chemotherapeutic agents, in general, have proven to be mitotoxic in various cell types, including renal and neuronal cells, leading to kidney impairment and chemotherapy-induced peripheral neuropathy (CIPN) through mitochondrial dysfunction (2). This dysfunction manifests as compromised adenosine triphosphate (ATP) and reactive oxygen species (ROS) production, the release of cytochrome c, and the opening of a redox-sensitive channel called the mitochondrial permeability transition pore (mPTP), which consequently causes a drop in mitochondrial membrane potential (MMP) and eventually leads to cell death (3, 4).

In studies regarding vincristine-induced peripheral neuropathy (VIPN) in the spinal cords of mice, it has been concluded that VCR treatment leads to a considerable rise in ROS and malondialdehyde (MDA) levels, resulting in oxidative stress and lipid peroxidation (LPO), respectively (5-7). Lipid peroxidation, a biochemical reaction, precipitates changes in plasma membrane structure and permeability (8-10). Vincristine also triggers cytochrome c release from mitochondria and a substantial decrease in Bcl-2, an anti-apoptotic protein, thereby facilitating apoptosis (5-7). Furthermore, VCR impacts another member of the Bcl-2 family, known as Bax, which serves as a pro-apoptotic factor. The onset of the apoptotic process within cells is believed to be linked to an elevated ratio of Bax to Bcl-2 (11-13). Even at low concentrations, VCR can lead to mitochondrial transport dysfunction and the upregulation of proteins implicated in ATP synthesis and oxidative phosphorylation (14, 15).

Vincristine exposure has been shown to decrease glutathione (GSH) concentrations in cells as a consequence of oxidative stress (16) and to cause a notable decline in the expression of succinate dehydrogenase (SDH) in Ramos cells. The expression levels of SDH can function as an oxidative damage biomarker, where heightened expression is associated with a diminished buildup of ROS (17). The administration of VCR leads to an increase in the size and total volume of lysosomes, causing lysosomal membrane permeabilization and destabilization, which ultimately results in apoptotic cell death (18). In 1981, it was demonstrated that following VCR and vinblastine (VBL) treatment, crystalline and paracrystalline structures appeared in the lysosomes of tubular cells in the kidneys of mice. These structures were deemed to result from autophagy induced by VCR and VBL and mitochondrial aggregation in autophagosomes (19). The necrotic effect of VCR on renal proximal tubular cells (RPTCs) in mice and dogs, causing damage to the brush borders, has been elucidated (20, 21). In 2019, necrosis of both proximal and distal tubules was reported, with a range of mechanisms implicated, including reduced renal antioxidant activities involving GSH enzymes, upregulation of Bax, downregulation of Bcl-2, elevated MDA levels, and decreased ATP and MMP, all of which play pivotal roles (1). Other investigations have confirmed these mechanisms as well (22, 23).

Oxidative stress occurs when there is an imbalance between the generation of free radicals, often exacerbated by mitochondrial dysfunction, and the compromised capacity of antioxidant defenses (24, 25). The association between mitochondrial dysfunction and a wide range of pathological changes and diseases has been well-documented (26). Conditions such as diabetes, cardiovascular diseases, renal disorders, neurodegenerative diseases, various types of cancer, and even aging are all linked to faulty mitochondria (27). Mechanistically, mitochondrial dysfunction is characterized by a substantial loss of ATP and MMP, along with an overproduction of ROS and other reactive species (28).

Potential therapeutic interventions aimed at restoring mitochondrial function could focus on increasing ATP production, enhancing antioxidant scavenging capacity, and preserving mitochondrial homeostasis within the renal system. This may be achieved by transferring freshly isolated mitochondria to affected cells (24, 25). Mitochondria exhibit a unique mechanism of fission and fusion, continually merging through fusion and dividing through fission. This dynamic process allows healthy mitochondria to fuse with damaged ones, eliminating impaired areas to preserve mitochondrial functionality through mitophagy (29, 30).

Mitochondrial transplantation involves introducing functional external mitochondria into cells compromised by mitochondrial defects, with the goal of restoring cellular viability and preventing further disease progression (28). The foundation of this concept originates from the work of Clark and Shay in 1982, who first demonstrated the capability of mammalian cells to uptake isolated mitochondria through endocytosis (31).

Previous research has shown that treating Parkinson’s model cells with mitochondria resulted in considerable improvements, including increased ATP and GSH levels, reduced ROS levels, and prevention of apoptosis (27). Another study highlighted the role of mitotherapy in improving cognitive and motor performance in aged mice by reducing oxidative stress (28). Hayashida et al. demonstrated that mitochondrial transplantation significantly alleviated ischemia-reperfusion injury (IRI) in specific tissues (32). Similarly, McCully et al. reported that replacing damaged mitochondria due to ischemia revitalized cellular functionality and reconstructed mitochondrial DNA (mtDNA) (33). Notably, only damaged cells uptake transplanted mitochondria, as healthy cells already possess functional mitochondria (34).

Research on the positive effects of mitochondrial therapy in drug-induced toxicity has shown that mitotherapy can mitigate liver toxicity associated with acetaminophen (35). In 2022, Hernandez-Cruz et al. suggested that mitotherapy may serve as a viable intervention for cadmium (Cd)-induced kidney injury stemming from mitochondrial dysfunction (36). Acute kidney injury (AKI) is another condition linked to mitochondrial impairment, and mitotherapy has proven to be a promising treatment to reduce apoptosis in renal tubular cells triggered by IRI (37).

## 2. Objectives

The present research, therefore, explores the cellular mechanisms underlying the nephrotoxicity of VCR, a topic that has received limited attention in previous literature, while also elucidating the protective role of transplanting isolated mitochondria from rat kidneys in restoring VCR-affected RPTCs.

## 3. Methods

### 3.1. Chemicals

All the chemicals utilised in this experiment were procured with the highest possible quality from Merck and Sigma companies (Germany).

### 3.2. Animals

All the animals used in our experiments (male Wistar rats) were purchased from the Pasteur Institute (Tehran, Iran), weighing between 200 - 230 grams and aged 5 - 9 weeks. They were maintained at a temperature of 20 - 22°C with a humidity level of 50 - 60% and a 12-hour light/dark cycle. After purchase, the male rats were allowed to acclimate to their environment for approximately one and a half weeks before the experiments commenced. All experimental procedures were reviewed and approved by the Ethical Committee for the Treatment of Experimental Animals at Iran University of Medical Sciences (IR.IUMS.AEC.1401.034) and were conducted in accordance with the guidelines of this committee. The RPTCs were isolated from male Wistar rats (38, 39).

### 3.3. Isolation and Preparation of RPT Cells

Renal proximal tubular cells isolation was carried out based on enzymatic methods (38, 39). The Wistar rats were anesthetized using 40 mg/kg ketamine and 10 mg/kg xylazine, and at the end of the experiments, they were euthanized via cervical dislocation. The kidneys were perfused with Ca-free Hanks' balanced salt solution (HBSS) containing 0.5 mM EGTA. The tissue was then digested with collagenase type II in HBSS containing 4 mM CaCl₂ and 1% penicillin-streptomycin. The kidney cortices were decapsulated and cut into 0.5 mm thick pieces. The RPTCs were obtained by passing the mixture through 120 µm and 60 µm mesh filters, respectively. Earl’s solution (pH 7.4) was used to wash and pellet 10⁶ cells/mL of RPTCs before resuspension. This process was conducted in round-bottom containers rotating in a 37°C water bath. Finally, the cells were suspended in 28 mM HEPES solution and incubated under a gas mixture of 10% O₂, 85% N₂, and 5% CO₂.

### 3.4. Evaluation of Cellular Viability

Cell toxicity was assessed by measuring the release of lactate dehydrogenase (LDH) using LDH assay kits from Sigma-Aldrich. A 10 µL sample was mixed with 1 mL of the indicator solution, and the absorbance was recorded at 37°C in 30-second intervals over a period of 4 minutes. A relative factor was applied to convert the absorbance rate at 340 nm to enzyme activity units. The LDH activity for all treated groups was expressed as µM per minute per liter (40).

### 3.5. Evaluation of Mitochondrial Functionality

In this study, differential ultracentrifugation (Hettich, Universal 320R, Tuttlingen, Germany) was used to isolate fresh and active mitochondria from the kidneys of Wistar rats (41, 42). The kidneys were extracted and minced in a cold isolation buffer containing mannitol, EDTA, and sucrose. Homogenization was performed using a glass homogenizer, and the homogenate was centrifuged at 4°C for 10 minutes to remove damaged cells and nuclei. Following this, 250 µL of bovine serum albumin (BSA) solution was added to the supernatant, which was then filtered through 40 µm and 5 µm filters, respectively. The filtrate was centrifuged at 10,000 × g for 10 minutes to pellet the mitochondria.

The mitochondrial pellet was resuspended and centrifuged again at 10,000 × g for 10 minutes. The final mitochondrial suspension was prepared in a homogenizing buffer containing Tris-HCl (0.05 M), MgCl₂ (2.0 mM), KCl (20 mM), sucrose (0.25 M), and Na₂HPO₄ (1.0 mM) at 4°C with a pH of 7.4. The concentration of mitochondrial proteins (80 µg/mL) was determined using the Coomassie Blue protein-binding assay with BSA as a standard (43). All subsequent mitochondrial assessments were normalized to samples containing 0.5 mg/mL of protein. The activity of mitochondrial SDH, a marker of mitochondrial function, was evaluated using MTT reduction at 570 nm with an ELISA reader (Tecan, Rainbow Thermo, Männedorf, Switzerland) (44).

### 3.6. Experimental Arrangement

The concentration of 10⁶ cells/mL of RPTCs was suspended in Earle's solution (pH = 7.4) after adding 1.5 mL of VCR at 37°C for 2 hours. The process of isolating fresh mitochondria from rat kidneys and their subsequent dilution to the required doses was carried out at 4°C. The RPTC medium was then replaced with one containing mitochondria and was kept in round-bottomed containers rotating in a water bath at 37°C for 4 hours.

### 3.7. Measurement of Succinate Dehydrogenase Activity in Renal Proximal Tubular Cells

This method is based on the color change of yellow tetrazolium powder to insoluble purple-black formazan crystals, which involves mitochondrial SDH. In this study, the reduction of MTT by SDH in the presence of both VCR (200 µM) and isolated mitochondria (80 µg/mL) was measured using an ELISA reader at 570 nm (44).

### 3.8. Measurement of Reactive Oxygen Species Concentrations

The rate of ROS production in RPTCs after mitochondrial transplantation was quantified by adding 1.6 µM of diacetyldichlorofluorescein (DCFH-DA) to the cells. Upon penetration into RPTCs, DCFH-DA undergoes hydrolysis to form non-fluorescent DCFH, which reacts with ROS to produce fluorescent dichlorofluorescein (DCF). The fluorescent intensity of DCF, indicative of ROS production, was measured at excitation and emission wavelengths of 500 nm and 520 nm, respectively, using a spectrofluorometer (Shimadzu RF5000U). The results were reported as fluorescence intensity units per 10^6^ cells (45).

### 3.9. Assessment of Mitochondrial Membrane Potential in Renal Proximal Tubular Cells

Mitochondrial membrane potential analysis was performed using Rhodamine 123 (Rh123), a cationic fluorescent dye. A 0.5 mL suspension of RPTCs was centrifuged, and the resulting pellet was re-suspended in 2 mL of medium containing 1.5 µM Rh123, followed by incubation at 37°C for 10 minutes. The Rh123 content in the RPTC suspensions was determined by measuring fluorescence using a spectrofluorometer (Shimadzu RF5000U) at excitation and emission wavelengths of 490 nm and 520 nm, respectively. The fluorescent intensity of Rh123 was calculated based on the fluorescence differences between Rh123 in control and treated groups (46).

### 3.10. Investigation of Lipid Peroxidation in Renal Proximal Tubular Cells

Lipid peroxidation, as an oxidative stress marker, was evaluated by measuring malondialdehyde (MDA) levels. The MDA produced in the sample was quantified by measuring the absorbance of the supernatant at 532 nm using an ELISA reader (Tecan, Rainbow Thermo) (47).

### 3.11. Measurement of Glutathione level in Renal Proximal Tubular Cells

After the addition of 0.5 mL of 10% trichloroacetic acid (TCA), the RPTCs were centrifuged at 11,000 rpm for 2 minutes. Subsequently, 0.5 mL of the obtained supernatant was diluted with 4.5 mL of EDTA buffer, and 1 mL of this mixture was added to 2.8 mL of phosphate-EDTA buffer. Finally, 100 µL of ortho-phthalaldehyde (OPA) solution was added. The resulting mixture was incubated at 25°C for 15 minutes, and the fluorescence absorbance was measured at excitation and emission wavelengths of 350 nm and 420 nm, respectively, using a Shimadzu RF5000U spectrofluorometer (48).

### 3.12. Evaluation of Lysosomal Membrane Damage

This research employed acridine orange to assess lysosomal membrane destabilization. As a weak base, acridine orange accumulates in lysosomes through proton trapping, producing red fluorescence at high concentrations within intact lysosomes and green fluorescence at lower concentrations in the cytosol and nucleus. The RPTCs were treated for 4 hours based on the designated groupings, followed by washing with phosphate-buffered saline (PBS) and a subsequent 20-minute incubation with acridine orange at 37°C in a dark environment. The RPTCs stained with acridine orange were subjected to centrifugation at 1,000 × g for 1 minute to separate them from the culture medium. The cells were subsequently washed twice with PBS to eliminate any residual acridine orange. Finally, the fluorescence intensity of the cell suspensions was assessed using a fluorescence spectrophotometer at excitation and emission wavelengths of 495 nm and 530 nm, respectively (49).

### 3.13. Adenosine Triphosphate Level Assay

The pre-incubation of 10^6^ cells/mL of VCR-treated RPTCs with 5-(N-ethyl-N-isopropyl) amiloride (EIPA) (100 µM) (50), cytochalasin D (10 µM) (51), and methyl-β-cyclodextrin (1 mM) (52) was performed to evaluate the process of mitochondrial uptake. The pre-incubation was carried out in specific flasks for 30 minutes. The isolated mitochondria (240 µg/mL) were then added to each flask and subsequently incubated at 37°C with 5% CO₂ for 4 hours. Finally, the inhibitory effects of mitochondrial transplantation on ATP levels were assessed (53).

### 3.14. Statistical Analysis

The outcomes were presented as mean ± SD, with one-way ANOVA used as the primary method of analysis, followed by post hoc tests. Additionally, GraphPad Prism 9 (GraphPad Software, La Jolla, CA) was utilized for both statistical and graphical analyses, with a significance level set at P < 0.05.

## 4. Results

### 4.1. The Evaluation of Mitochondrial Functionality in Isolated Mitochondria

**Figure 1.**
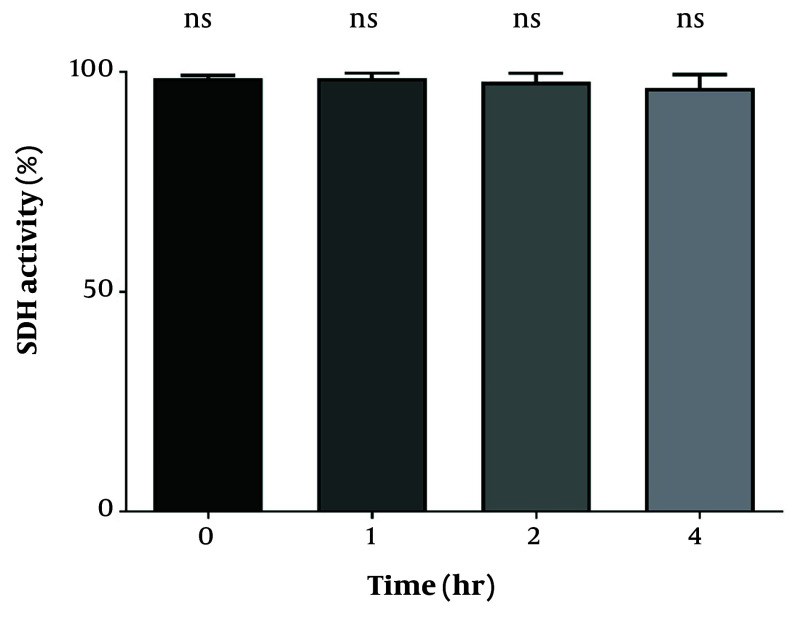
Mitochondrial functionality. No considerable difference in succinate dehydrogenase (SDH) activity compared to time zero.

To assess the proper function of isolated mitochondria, the MTT assay was performed at 570 nm to measure SDH activity in the mitochondria. As shown in Figure 1, SDH activity did not exhibit any significant differences during the incubation periods of 1, 2, and 4 hours.

### 4.2. The Cytotoxic Effect of Vincristine on Renal Proximal Tubular Cells and Determination of IC50 for Vincristine

**Figure 2.**
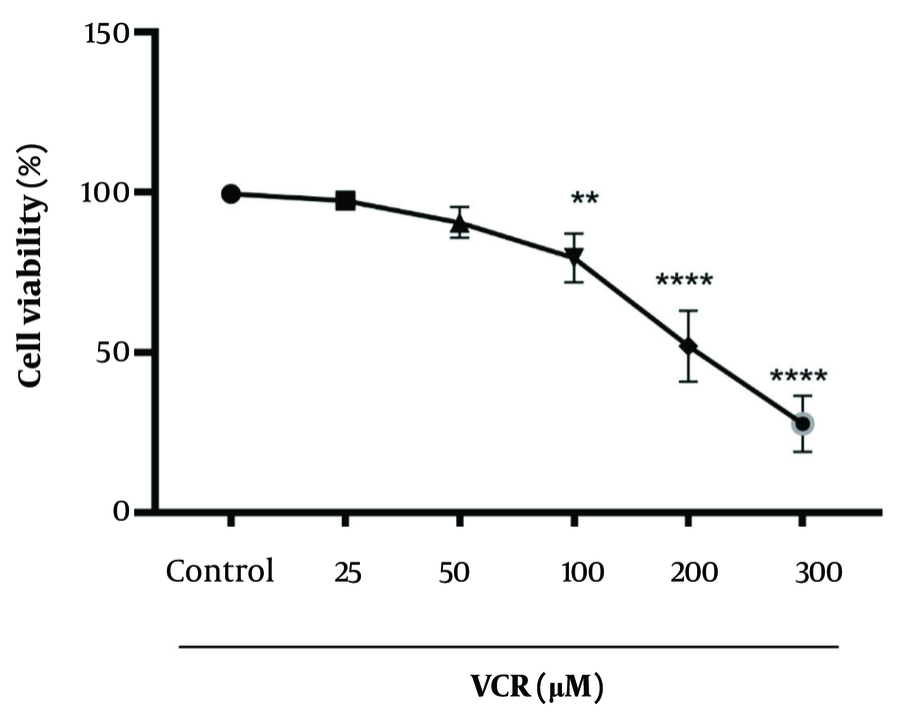
Vincristine (VCR) cytotoxicity and its IC_50_ determination. The IC_50_ for VCR was considered to be 200 µM. Values were depicted as mean ± SD (n = 5). **P < 0.01 and ****P < 0.0001 in comparison to control.

A significant reduction (P < 0.0001) in cell viability was observed in rat RPTCs after a 2-hour incubation with 200 µM VCR, as shown in Figure 2. 

### 4.3. Optimal Protective Concentration of Mitochondrial Transplantation Against Vincristine -Induced Renal Proximal Tubular Cells Damage

**Figure 3.**
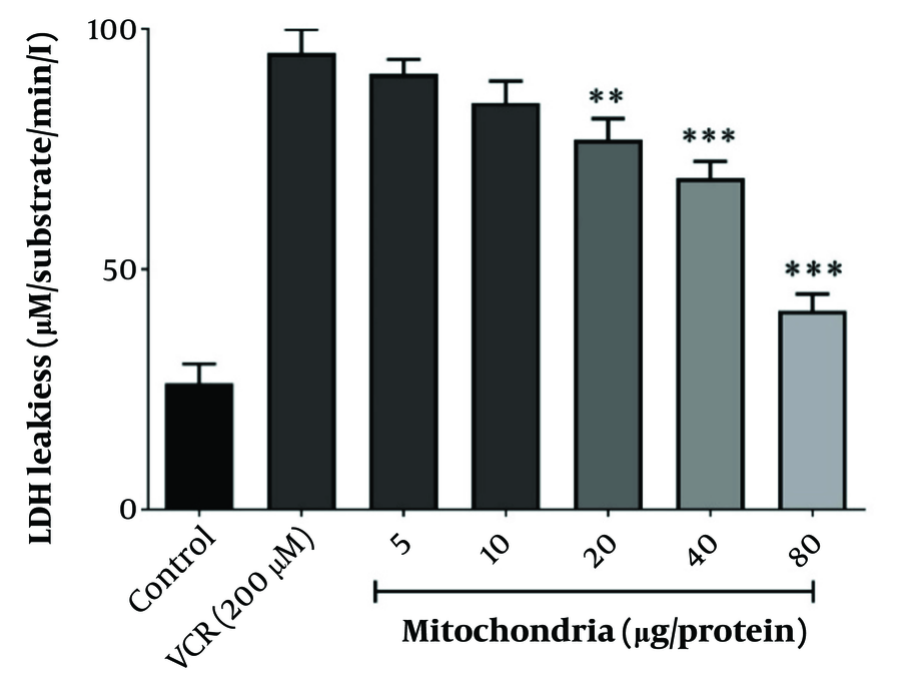
The best protective concentration of isolated mitochondria against VCR-induced cytotoxicity. The best protective concentration of isolated mitochondria against VCR-induced cytotoxicity was considered to be 80 µg/mL. Values were depicted as mean ± SD (n = 5). **P < 0.01 and ***P < 0.001 in comparison to control.

Lactate dehydrogenase leakage, indicative of VCR-induced cytotoxicity, was prevented by co-incubating RPTCs with 80 µg/mL of freshly isolated mitochondria, as illustrated in Figure 3. Therefore, 80 µg/mL was considered the optimal protective concentration of isolated mitochondria against VCR-induced RPTC damage.

### 4.4. The effect of Mitotherapy on Restoring Succinate Dehydrogenase Activity

**Figure 4.**
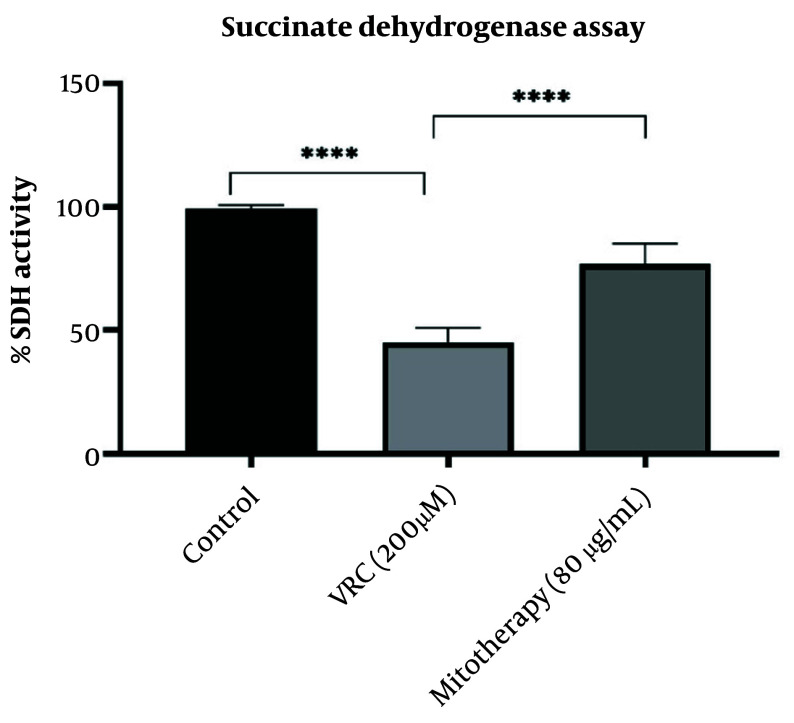
The effect of mitotherapy on restoring succinate dehydrogenase (SDH) activity. The SDH activity decreased in vincristine (VCR) group in comparison to control group (****P < 0.0001) whereas, mitotherapy increased SDH activity in comparison to VCR group (****P < 0.0001). Values were depicted as mean ± SD (n = 5).

Vincristine application impaired SDH activity, which was measured using the MTT assay, where MTT is reduced to purple formazan at 570 nm. As reported in Figure 4, mitotherapy significantly restored SDH activity in RPTCs (P < 0.0001).

### 4.5. The Aftermath of Mitotherapy on Reactive Oxygen Species Generation

**Figure 5.**
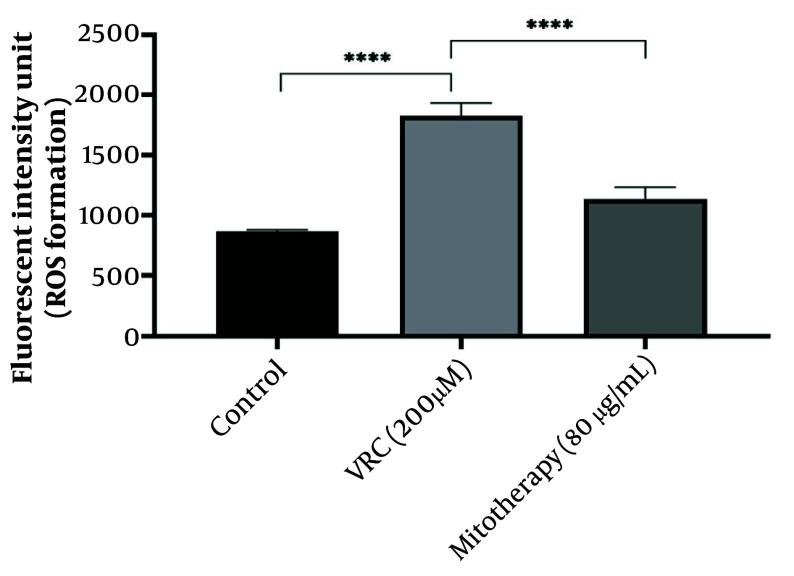
The response of reactive oxygen species (ROS) generation to mitotherapy. The amount of ROS generation increased in vincristine (VCR) group in comparison to control group (****P < 0.0001) whereas, mitotherapy decreased ROS generation in comparison to VCR group (****P < 0.0001). Values were depicted as mean ± SD (n = 5).

According to the results obtained (Figure 5), VCR (200 µM) significantly (P < 0.0001) increased ROS levels in cells compared to the control group. Mitochondrial transplantation (80 µg/mL) significantly reduced ROS levels in VCR-treated RPTCs (P < 0.0001), indicating that isolated mitochondria can mitigate oxidative damage caused by VCR.

### 4.6. The Effect of Mitotherapy on Mitochondrial Membrane Potential Collapse in Renal Proximal Tubular Cells

**Figure 6.**
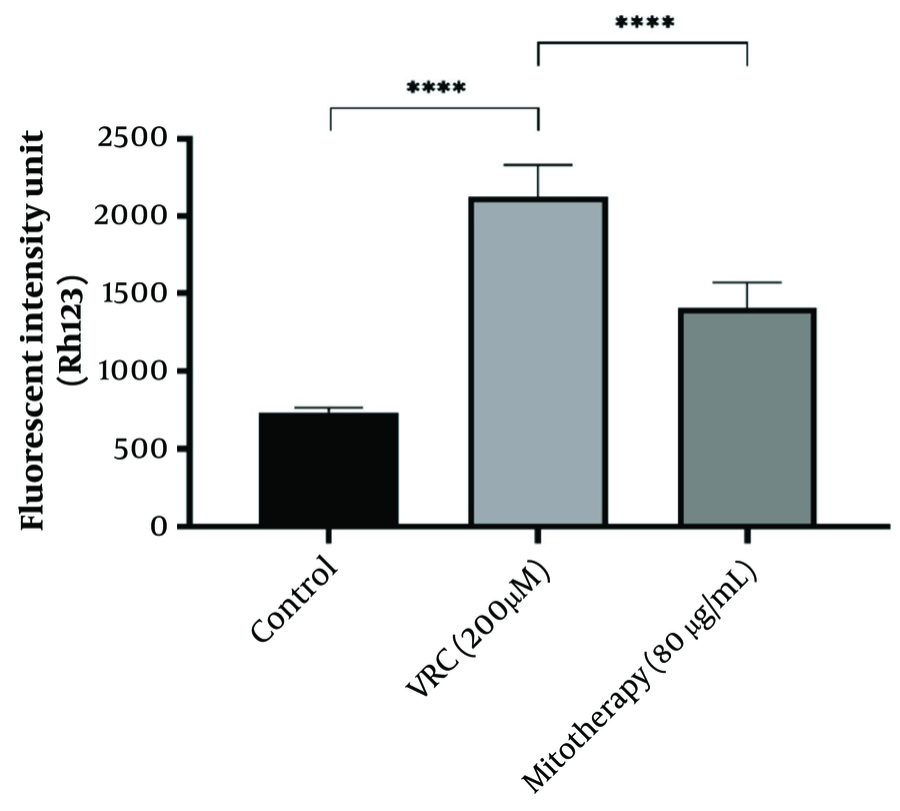
The effect of mitotherapy on mitochondrial membrane potential (MMP) collapse in renal proximal tubular cells (RPTCs). The drop in MMP was significant in vincristine (VCR) group compared to control group (****P < 0.0001) whereas, mitotherapy prevented MMP collapse compared to VCR group (****P < 0.0001). Values were depicted as mean ± SD (n = 5).

The MMP collapse is considered one of the causes of mitochondrial dysfunction. As shown in Figure 6, VCR (200 µM) induced a significant collapse in MMP compared to the control group. Mitochondrial transplantation (80 µg/mL) demonstrated a significant protective effect against VCR-induced MMP collapse in RPTCs (P < 0.0001).

### 4.7. The Reducing Impact of Mitotherapy on Lysosomal Damage

**Figure 7.**
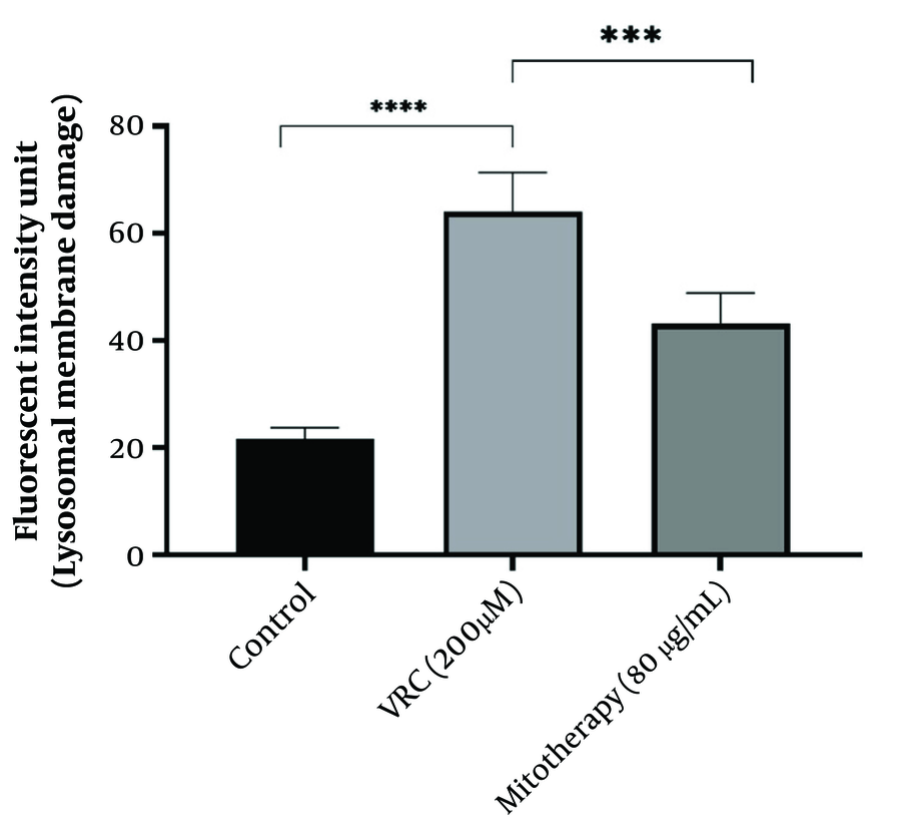
The reducing impact of mitotherapy on lysosomal damage. The damage to lysosomal membrane was significant in vincristine (VCR) group compared to control group (****P < 0.0001) whereas, mitotherapy decreased lysosomal damage compared to VCR group (***P < 0.0001). Values were depicted as mean ± SD (n = 5).

As indicated in Figure 7, lysosomal damage was also a consequence of VCR toxicity. The VCR at a concentration of 200 µM caused significant lysosomal membrane damage (P < 0.0001) in RPTCs, while subsequent mitochondrial transplantation (80 µg/mL) significantly reduced this lysosomal damage (P < 0.0001).

### 4.8. The Enhancing Effect of Mitotherapy on Glutathione Levels

**Figure 8.**
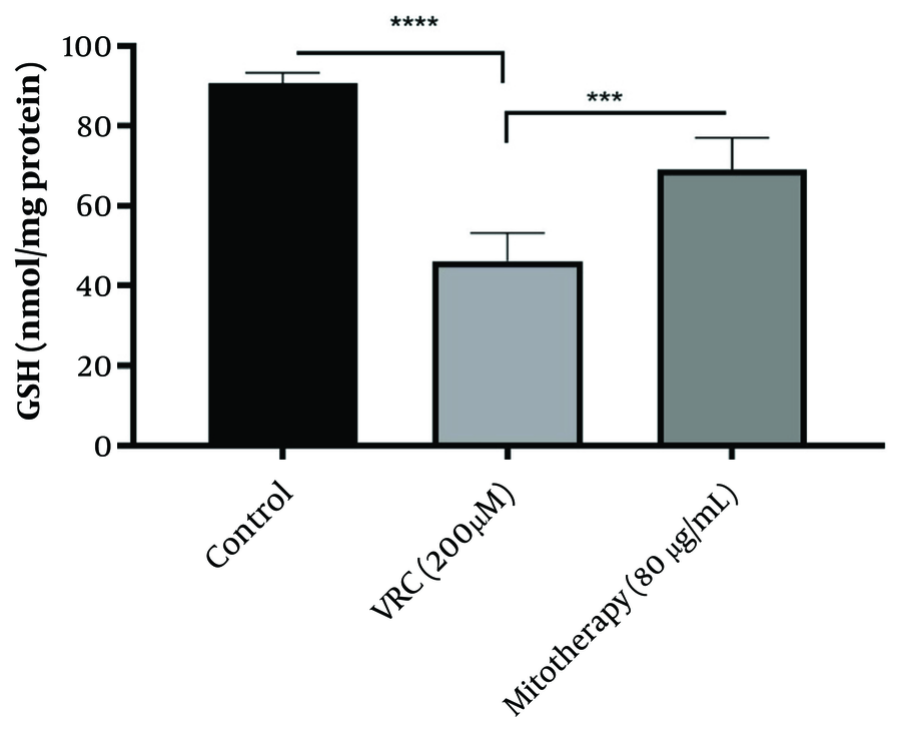
The enhancing effect of mitotherapy on glutathione (GSH) levels. The GSH levels were reduced in vincristine (VCR) group compared to control group (****P < 0.0001) whereas, mitotherapy increased GSH levels compared to VCR group (***P < 0.0001). Values were depicted as mean ± SD (n = 5).

The results obtained (Figure 8) indicated that VCR (200 µM) significantly reduced GSH levels in RPTCs (P < 0.0001), which is crucial in initiating oxidative stress. Additionally, mitochondrial transplantation (80 µg/mL) substantially improved GSH levels in RPTCs (P < 0.0001).

### 4.9. The Reducing Effect of Mitotherapy on Lipid Peroxidation

**Figure 9.**
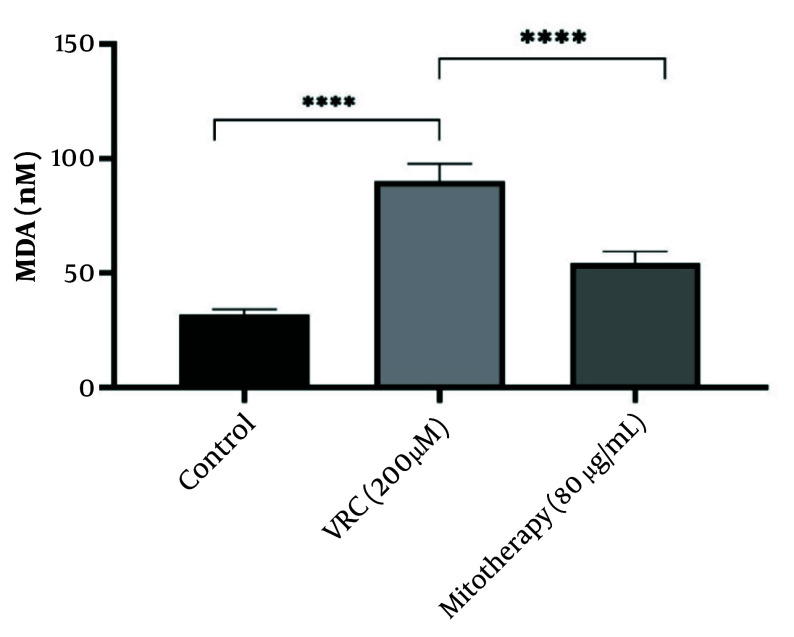
The reducing effect of mitotherapy on lipid peroxidation. The MDA levels were increased in vincristine (VCR) group compared to control group (****P < 0.0001) whereas, mitotherapy reduced the MDA levels compared to VCR group (****P < 0.0001). Values were depicted as mean ± SD (n = 5).

The results shown in Figure 9 indicate that VCR (200 µM) significantly increased MDA levels in RPTCs (P < 0.0001). Mitochondrial transplantation (80 µg/mL) was subsequently found to considerably reduce MDA levels and lipid peroxidation in RPTCs (P < 0.0001).

### 4.10. Determination of the Uptake Mechanism of Transplanted Mitochondria into Renal Proximal Tubular Cells

**Figure 10.**
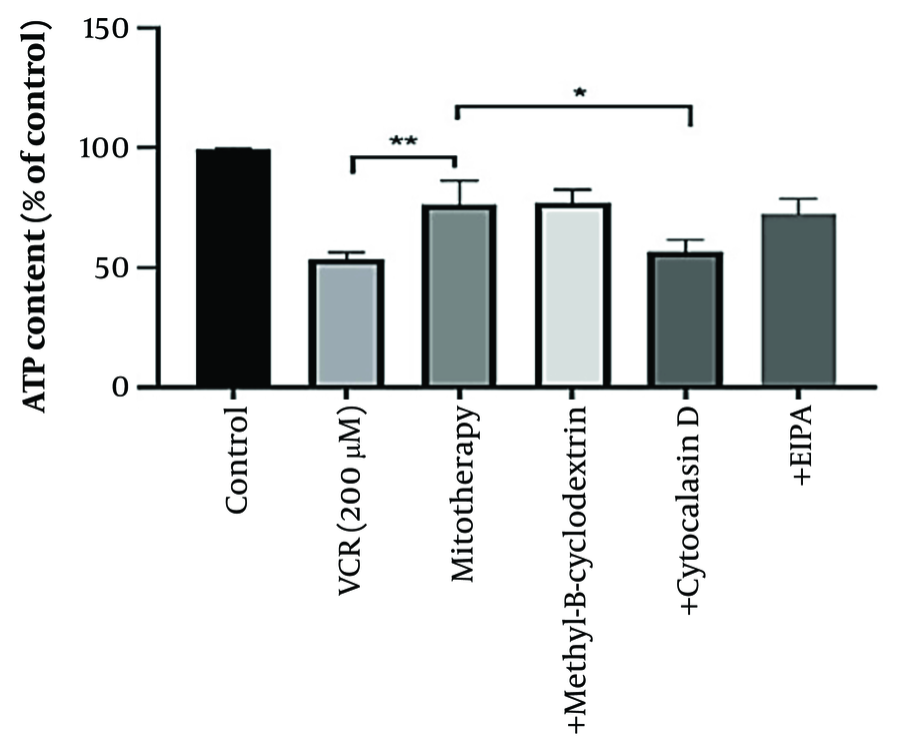
Determination of mitochondrial uptake mechanism. Cytochalasin D prevented the protective effects of mitotherapy on adenosine triphosphate (ATP) indicating the internalization mechanism of isolated mitochondria. Values were depicted as mean ± SD (n = 5). **P < 0.01 vincristine (VCR) group vs mitotherapy and *P < 0.05 mitotherapy vs cytochalasin D group.

According to our results, pre-incubation with cytochalasin D reduced the protective effects of mitochondrial transplantation, indicating the involvement of an internalization mechanism for isolated mitochondria. As illustrated in Figure 10, ATP content did not improve with mitotherapy following cytochalasin D treatment. Additionally, VCR significantly reduced ATP content in the cells (P < 0.0001).

## 5. Discussion

This study aims to analyze the mitochondrial toxicity associated with VCR and to determine the impact of mitotherapy in mitigating the toxic effects of VCR on RPTCs.

Chemotherapy commonly causes proximal tubular impairment through mitotoxicity. Given that a considerable amount of VCR is excreted unchanged via this part of the kidneys, the importance of studying VCR-induced nephrotoxicity becomes even more prominent (1). Previous studies have indicated that the nephrotoxic effects of VCR primarily stem from a disruption in redox balance. This imbalance triggers several pathological events, including elevated levels of ROS and MDA, diminished ATP synthesis, a reduction in MMP, and decreased activities of SDH and GSH. Collectively, these factors contribute to oxidative damage and ultimately result in cellular apoptosis.

Earlier investigations have also revealed the profound effect of VCR on Bcl-2 family members, as it suppresses Bcl-2, an anti-apoptotic protein, and promotes Bax, a pro-apoptotic protein. The release of cytochrome c into the cytosol is another indication of VCR-induced oxidative stress (5-7). Upon administration of VCR, there is a notable increase in the size and total volume of lysosomes, which results in the destabilization and permeabilization of their membranes, thereby leading to cell death via apoptosis (18).

The elevated production of ROS from compromised mitochondria can affect the integrity of the lysosomal membrane, potentially leading to the release of lysosomal contents into the cytoplasm. Furthermore, research indicates that these reactive species can activate phospholipase A2 (PLA2), resulting in the destabilization and increased permeability of the lysosomal membrane (54-56). The interaction of free radicals with intra-lysosomal free iron facilitates the production of highly reactive hydroxyl radicals through a Fenton-type reaction. This mechanism plays a key role in triggering lysosomal membrane permeabilization (LMP) by promoting ongoing lipid peroxidation of lysosomal membranes, which leads to the formation of lipofuscin and further damage to lysosomal membrane proteins (57, 58). Alternatively, lysosomal membrane permeability may be induced prior to any mitochondrial impairment, as lysosomal enzymes can target the mitochondrial membrane and promote the formation of ROS. This, in turn, could further increase the vulnerability of the lysosomal membrane to damage (59).

The kidneys are characterized by a high mitochondrial content, second only to that of the heart, which is essential for meeting the energy demands of various cellular processes. During these processes, especially ATP production, the emergence of reactive species, notably ROS, is unavoidable (24, 25). The generation of ROS during oxidative phosphorylation to produce ATP is significantly influenced by mitochondria. The mitochondrial membrane potential serves as a regulatory factor for the rate of ROS production within these organelles. However, excessive accumulation of ROS may induce sustained opening of the mPTP, culminating in a surge of ROS and the risk of oxidative stress and mitochondrial injury. This mitochondrial damage results in the release of cytochrome c, loss of ATP and MMP, downregulation of Bcl-2, and an increase in ROS levels (60, 61). It is well-established that any form of mitochondrial dysfunction is responsible for the pathophysiology of renal injury (26). Oxidative injury is considered one of the important causes of renal fibrosis, as any renal abnormality could lead to excessive generation of ROS (25, 62).

Since mitochondrial dysfunction is a fundamental mechanism in drug-induced kidney toxicity, mitotherapy can be used to replace inefficient mitochondria with healthy ones. In a study conducted by Arjmand et al. in 2022, the effect of transplanting freshly isolated mitochondria on gentamicin-induced toxicity in RPTCs was investigated (63). In another research endeavor, the influence of freshly isolated mitochondria on the toxicity of favipiravir in RPTCs was evaluated. The results of the statistical analysis indicated that the introduction of healthy mitochondria significantly reduced cellular toxicity, ROS generation, MMP disruption, lysosomal injury, GSH depletion, and caspase-3 activation caused by favipiravir. Additionally, this intervention led to an increase in ATP production, Bcl-2 expression, and the ratio of GSH/GSSG in RPTCs (64). 

In a comparable research study focusing on the drugs ifosfamide and doxorubicin, it was demonstrated that healthy mitochondria can be efficiently taken up by RPTCs, and their incorporation alleviates the cytotoxicity associated with oxidative damage from these agents in rat kidney tubular cells (65, 66). Thus, mitochondrial transplantation represents a potent therapeutic option for mitigating kidney toxicity resulting from chemical substances.

Ischemia-reperfusion (I/R) is a contributing factor to mitochondrial malfunction, which is associated with significant kidney injuries, including acute kidney injury (AKI) and chronic kidney disease (CKD). This malfunction can lead to elevated levels of ROS and malondialdehyde (MDA), while simultaneously reducing ATP production and MMP. Additionally, the antioxidant capacities of superoxide dismutase (SOD) and GSH are significantly compromised, leading to redox imbalance. The loss of MMP triggers the opening of mitochondrial permeability transition (MPT) pores, resulting in the release of cytochrome c into the cytosol. The decreased ratio of Bcl-2 to Bax ultimately leads to cell death (67). Mechanisms such as chemotherapy and I/R have been implicated in tubular cell death, which is associated with the generation of mitochondrial ROS and the opening of MPT pores (68). 

Falone et al. hypothetically linked the apoptotic death of cancerous cells to mitochondrial impairment, manifested by ROS release, cytochrome c discharge from the cells, and a notable loss of ATP and MMP (69). Therefore, replacing damaged mitochondria with functional ones could therapeutically address these issues (67). Mitochondrial transplantation (MT) is an emerging experimental method that has shown promising outcomes in addressing mitochondrial abnormalities linked to cardiac and kidney dysfunction, predominantly influenced by oxidative stress (27, 70). 

Evidence from animal studies has demonstrated that restoring mitochondrial function and preventing apoptosis in cardiomyocytes are some of the notable benefits of mitotherapy. Direct introduction of isolated mitochondria into the myocardium affected by ischemia-reperfusion injury was conducted by the McCully group, revealing significant improvement in ventricular function within a few days (71, 72). The incorporation of mitochondria leads to a reduction in oxidative damage, as evidenced by decreased lipid peroxidation products within the lesion areas of cardiomyocytes (73). Additionally, research by other investigators has elucidated the therapeutic effects of delivered mitochondria in the ischemic heart, including reduced ROS production, prevention of apoptosis, and increased ATP content (24, 71, 74). 

Findings from previous studies suggest that intra-arterial injection of mitochondria serves as an effective strategy for protecting the kidneys from I/R injury, significantly improving renal function and mitigating renal damage (75).

In a diabetic nephropathy model, the administration of mitochondria effectively abolished ROS production by restoring the levels of superoxide dismutase 2 (SOD2) and preventing apoptosis through the upregulation of Bcl-2 protein. In another model of kidney damage, mitochondrial transplantation (MT) also promoted SOD and ATP levels while reducing apoptosis by increasing Bcl-2 expression (70). Additionally, a study involving the intravenous injection of mitochondria into the brains of Parkinson’s disease (PD) model mice demonstrated that mitochondrial transplantation suppressed PD progression by reducing free radical generation and decreasing apoptotic cells. Moreover, mitochondrial transfer has been introduced as a promising intervention to combat stroke, showing a significant reduction in cellular redox imbalance and apoptosis in a rodent ischemic brain model (76, 77). 

Our research began by confirming that actin-dependent endocytosis serves as the mechanism for mitochondrial internalization. We further substantiated that the toxicity of VCR is derived from an imbalance in redox processes. Our data demonstrated that exposure of RPTCs to VCR resulted in a significant increase in ROS and MDA, a considerable decrease in ATP and MMP, a notable reduction in SDH activity and GSH antioxidant capacity, as well as damage to the lysosomal membrane structure. These findings are consistent with prior research. 

**Figure 11.**
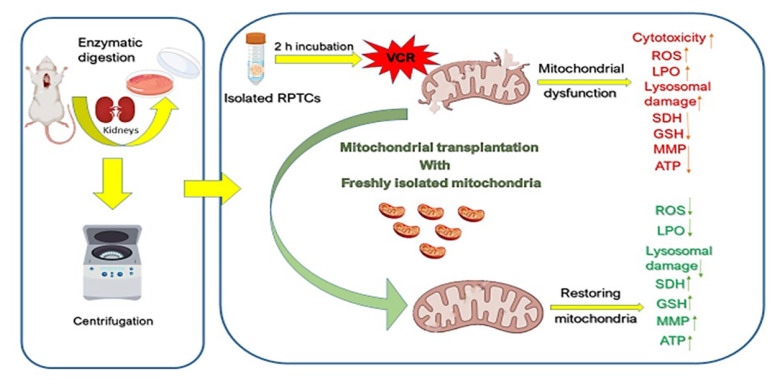
Schematic representation of vincristine (VCR)-induced nephrotoxicity through mitochondrial impairment and the protective effect of mitochondrial transplantation. Transferring the freshly isolated mitochondria into VCR-damaged renal proximal tubular cells (RPTCs) diminishes oxidative stress by reducing ROS and MDA formation, increasing succinate dehydrogenase (SDH) activity, GSH concentrations and adenosine triphosphate (ATP) levels.

Additionally, our data illustrated that the introduction of freshly isolated mitochondria to damaged RPTCs significantly reversed the aforementioned changes, corroborating the principles established in mitochondrial medicine research. In essence, the findings indicate that VCR is inherently nephrotoxic, and the application of mitotherapy may offer a beneficial therapeutic strategy. A schematic representation of the present study is depicted in Figure 11. 

### 5.1. Conclusions

This study presents the transplantation of freshly isolated mitochondria as a potential therapeutic approach for addressing kidney injury induced by VCR. The findings indicate that mitochondrial transfer mitigates VCR-related cytotoxic effects in rat RPTCs. Furthermore, this intervention reduces oxidative stress and preserves the integrity of both mitochondrial and lysosomal membranes, effectively preventing the activation of cell death signaling pathways triggered by VCR in rat RPTCs.

## Data Availability

The dataset presented in the study is available on request from the corresponding author during submission or after publication.
